# Stair descending exercise increases muscle strength in elderly males with chronic heart failure

**DOI:** 10.1186/1756-0500-6-87

**Published:** 2013-03-08

**Authors:** Anastasios A Theodorou, George Panayiotou, Vassilis Paschalis, Michalis G Nikolaidis, Antonios Kyparos, Lida Mademli, Gerasimos V Grivas, Ioannis S Vrabas

**Affiliations:** 1 Laboratory of Exercise, Health and Human Performance, Research Center, European University Cyprus, Engomi, Diogenes Str 6, Nicosia, Cyprus; 2 Department of Physical Education and Sport Sciences at Serres, Aristotle University of Thessaloniki, Agios Ioannis, Serres 62110, Greece; 3 Institute of Human Performance and Rehabilitation, Center for Research and Technology, Trikala, Thessaly, Greece; 4 Exercise Physiology and Biochemistry Laboratory, Department of Physical Education and Sport Sciences at Serres, Aristotle University of Thessaloniki, Serres, Greece

**Keywords:** Aging, Chronic heart failure, Eccentric exercise, Muscle strength, Sarcopenia

## Abstract

**Background:**

Previous studies from our group have shown that "pure" eccentric exercise performed on an isokinetic dynamometer can induce health-promoting effects that may improve quality of life. In order to investigate whether the benefits of "pure" eccentric exercise can be transferred to daily activities, a new and friendlier way to perform eccentric exercise had to be invented. To this end, we have proceeded to the design and construction of an automatic escalator, offering both stair descending (eccentric-biased) and stair ascending (concentric-biased) exercise.

**Findings:**

Twelve elderly males (60-70 yr) with chronic heart failure participated in the present study. Participants carried out six weeks of stair descending or ascending training on the novel SmartEscalator device. Muscle damage and performance indices were evaluated before and at day 2 post exercise at the first and sixth week of training. Both training regimes increased, albeit not significantly in some cases, eccentric, concentric and isometric torque. After six weeks of stair descending exercise, eccentric, concentric and isometric peak torque increased 12.3%, 7.7% and 8.8%, respectively, whereas after stair ascending exercise eccentric, concentric and isometric peak torque increased 7.1%, 9.6% and 5.9%, respectively.

**Conclusions:**

Stair descending exercise appears to be a pleasant and mild activity that can be easily followed by the elderly. Compared to the more demanding stair ascending exercise, changes in muscle strength are similar or even greater. Elderly or people with impaired endurance wishing to increase their muscle strength may be benefited by participating in activities with strong eccentric component, such as stair descending.

## Findings

### Background

The lack of regular exercise or physical inactivity is an underlying factor in the risk of developing cardiovascular diseases and many other chronic diseases [1]. Unfortunately, the vast majority of elderly and individuals suffering from cardiovascular diseases avoid exercise. In our opinion, the major reason why these individuals abstain from exercise is the belief that exercise requires special activities, too much effort has to be put on and require particular physical skills from the participants. Indeed, these concerns are true for most types of exercise.

**Figure 1:**
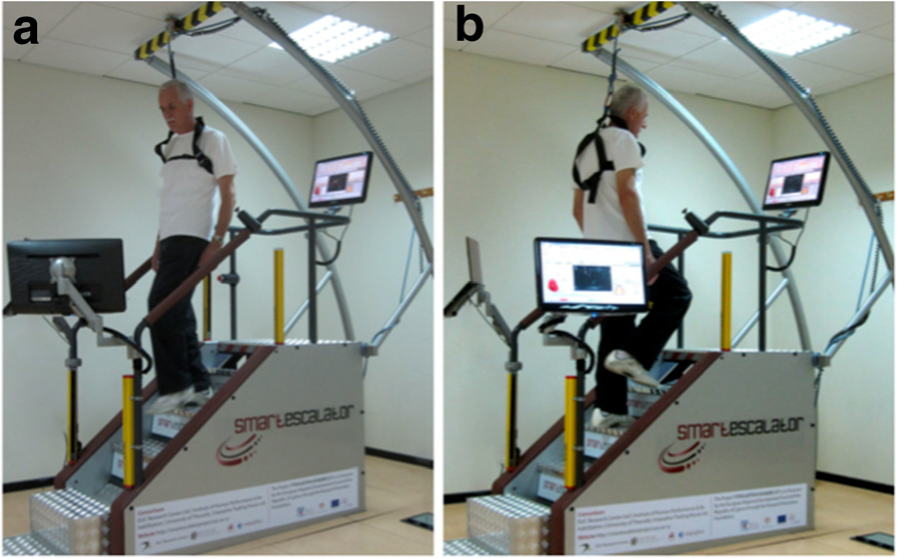
**The SmartEscalator device was invented, designed and constructed by our group and it is the first of its kind offering both stair descending (eccentric-biased) and stair ascending (concentric-biased) exercise.** The major advantage of the new device is the stair descending mode that trains individuals to walk down stairs. This downward movement offers simultaneously a strong eccentric stimulus that has been linked with health-promoting effects. The participant in the figure gave his written consent for the publication of this information and to be appear in the journal’s and associated publications.

 Therefore, modern training regimes should be efficient, short in duration and easier to be performed than traditional exercise interventions. In a previous study from our group, we reported that only 30 min of eccentric exercise per week for eight weeks was sufficient to improve health risk factors [2]. However, in that investigation, we used specialized equipment employing abnormal muscle movement (i.e., eccentric actions of knee extensors on isokinetic dynamometer) and we adopted an exercise protocol of maximal effort that is difficult to be followed by the elderly or people with restricted physical ability. Considering these limitations, and in order to investigate whether the benefits of pure eccentric exercise can be transferred to daily activities of life [2], a new and friendlier way to perform eccentric exercise had to be invented. To this end, we have proceeded to the design and construction of an automatic escalator, offering both stair descending (eccentric-biased) and stair ascending (concentric-biased) exercise (Figure 1). Therefore, the main aim of this pilot study was to compare the chronic effect of stair descending versus stair ascending exercise on muscle damage and performance in elderly individuals with chronic heart failure. We have chosen to perform the study in elderly people with chronic heart failure since the novel exercise device has been especially designed for individuals with impaired physical abilities such as elderly or people suffering from chronic diseases.

### Materials and methods

#### Design

**Figure 2:**
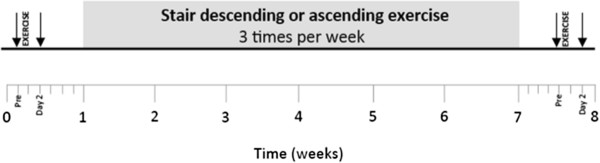
**Study design.** Downward arrows indicate the time of physiologic measurements and blood sampling.

An overview of the study design is shown in Figure 2. Participants were recruited after advertising the study in the local media. Then, physicians assessed the stage of heart failure according to the New York Heart Association (NYHA) functional classification system. Participants were allocated according to age, body mass index, and maximum isometric, eccentric and concentric torque into 2 equal-sized groups: a stair descending group (n = 6, age 66.8 ± 1.7 yr, body mass 85.8 ± 3.1 kg, body fat 29.7 ± 2.3%;mean ± SEM) and a stair ascending group (n = 6, age 64.8 ± 2.3 yr, body mass 82.1 ± 2.3 kg, body fat 31.6 ± 2.7%). The volunteers of both groups did not spend more than one hour per week on recreational activities (e.g., walking, cycling, dancing) the last six months before entering the study. In addition, the volunteers were instructed to record all their recreational activities through the experimental intervention with a validated self-administered questionnaire [3]. Two weeks before the beginning of the study, the volunteers visited the laboratories in which the exercise and measurements were taken place and familiarised with the relevant equipment. At the beginning of the study, the volunteers performed an acute bout of stair descending or stair ascending exercise on an automatic escalator designed and built by our group (4 sets of 3 min each, speed was set at 45 steps·min^-1^, in total 540 steps for both groups). During the stair descending exercise, the stairs were moving upwards and the participants were descending the escalator (Figure 1a), while during stair ascending exercise, stairs were moving downwards and the participants were ascending the escalator (Figure 1b). Step height was 20.5 cm. Before and at day 2 post exercise, physiologic measurements were performed and blood samples were collected. Then, participants carried out six weeks of stair descending or ascending training consisting of three exercise sessions per week. Each exercise session lasted for 12 min, separated in 4 sets of 3 min each, and there was a 2-min rest between each set. Heart rate was continuously monitored during exercise. The first two weeks, speed was set at 45 steps·min^-1^ (in total 540 steps for both groups), the next two weeks at 50 steps·min^-1^ (in total 600 steps for both groups) and the last two weeks at 55 steps·min^-1^ (in total 660 steps for both groups). Afterward, they repeated the acute stair descending or ascending protocol, as carried out at the beginning of the study, and the same physiologic measurements were performed and blood samples were collected. Exercise was conducted under the supervision of an exercise physiologist. The procedures were in accordance with the Helsinki declaration of 1975, as revised in 2000, and approval was received from the Ethics Committee of the European University Cyprus. Written informed consent to participate in the study was provided by all volunteers and their personal physicians.

#### Measurements

An isokinetic dynamometer (Cybex, Ronkonkoma, NY) was used for the measurement of isometric peak torque (at 90° knee flexion), concentric peak torque at 60°·s^-1^, and eccentric peak torque at 60°·s^-1^ of knee extensors. Five maximal voluntary contractions (MVC) were performed, and the best three were recorded. There was a 2-min rest between efforts. The subjects were verbally encouraged in every repetition to perform better than their previous effort. Each volunteered assessed delayed onset muscle soreness (DOMS) during a squat movement (90° knee flexion), and perceived soreness was rated on a scale ranging from 1 (normal) to 10 (very sore). The activity of creatine kinase (CK) has been measured in a Cobas Integra Plus 400 chemistry analyzer (Roche Diagnostics, Mannheim, Germany).

#### Statistical analysis

Differences on physical characteristics between the groups at baseline were examined by using an unpaired Student’s t test. A three-factor ANOVA [group (stair descending or ascending) × week of exercise (first week or sixth week) × time (before exercise and at day 2 post exercise)] was used to examine the effect of stair training on muscle damage and performance. If a significant interaction was obtained, pairwise comparisons were performed using the Sidak method (a = 0.05).

 To determine the meaningfulness of the effect of exercise, effect sizes were calculated as the difference between pre- and post-exercise (at week 1 and at week 6) and between baseline pre- and post training values (at week 1 and week 6) divided by the standard deviation of the pre-exercise values in each group (Table 1). According to a modified Cohen’s scale ( http://newstats.org), effect sizes of 0.2, 0.6, 1.2, 2.0, and 4.0 were considered to be small, moderate, large, very large, and nearly perfect, respectively. For comparison, the values of the original Cohen’s scale are 0.2 for small, 0.5 for moderate, and 0.8 for large effects.

**Table 1:** Muscle function and muscle damage indices at pre exercise and day 2 post exercise in the first and sixth week of stair descending and stair ascending groups (mean ± standard error of the mean)

	**Week 1**		**Week 6**		**Main effects and interactions**							**Effect sizes**		
	**Pre**	**Day 2**	**Pre**	**Day 2**	**G × W × T**	**G × W**	**G × T**	**W × T**	**G**	**W**	**T**	**Week 1 (pre vs. day 2)**	**Week 6 (pre vs. day 2)**	**Pre (week 1 vs. week 6)**
*Eccentric peak torque (Nm)*														
Descending	155.5 ± 10.1	132.3 ± 10.6^a^	174.5 ± 7.7	177.8 ± 8.6^b^	<.001	>.05	.004	>.05	>.05	>.05	>.05	0.94	-0.20	-0.77
Ascending	148.3 ± 12.0	158.7 ± 13.6	158.7 ± 11.9	156.8 ± 12.7								0.31	-0.24	-0.35
*Concentric peak torque (Nm)*														
Descending	125.3 ± 15.0	114.3 ± 13.5	135.2 ± 12.9	141.3 ± 10.1	>.05	>.05	>.05	>.05	>.05	>.05	>.05	0.31	-0.25	-0.26
Ascending	118.5 ± 13.7	122.5 ± 10.9	133.3 ± 12.0	137.8 ± 11.2								-0.11	-0.15	-0.44
*Isometric peak torque (Nm)*														
Descending	142.7 ± 10.0	125.3 ± 11.4^a^	154.5 ± 9.4	158.2 ± 12.1^b^	>.05	>.05	.009	<.001	>.05	>.05	>.05	0.71	-0.16	-0.51
Ascending	135.0 ± 10.7	139.2 ± 10.3	141.7 ± 9.6	152.7 ± 10.2								-0.17	0.46	-0.27
*Range of movement (*^*o*^*)*														
Descending	119.2 ± 0.8	116.2 ± 1.7^a^	119.3 ± 0.6	118.7 ± 1.0	>.05	>.05	>.05	>.05	>.05	>.05	.035	1.47	0.64	0.07
Ascending	118.2 ± 0.7	117.8 ± 1.0	119.5 ± 0.5	119.0 ± 0.8								0.21	0.59	-0.83
*DOMS (1-10)*														
Descending	1.0 ± 0.0	3.2 ± 1.0^a^	1.0 ± 0.0	1.8 ± 0.7	>.05	>.05	>.05	>.05	>.05	>.05	.009	NC	NC	NC
Ascending	1.0 ± 0.0	1.7 ± 0.5	1.0 ± 0.0	2.2 ± 0.8								NC	NC	NC
*CK (U/L)*														
Descending	80.3 ± 23.9	913.3 ± 237.5^a^	117.0 ± 24.4	243.5 ± 95.6^b^	>.05	>.05	>.05	>.05	.034	>.05	.041	-16.2	-0.76	-0.71
Ascending	99.7 ± 19.4	298.2 ± 88.3^a^	93.3 ± 20.1	121.2 ± 54.2								-4.25	-0.88	0.13

DOMS, delayed onset muscle soreness; G × W × T, three-way interaction for group, week, and time; G × W, two-way interaction for group and week; G × T, two-way interaction for group and time; W × T, two-way interaction for week and time; G, main effect of training group; W, main effect of week; T, main effect of time; NC, not calculated.

^a^ Significant difference from the pre exercise value in the same group.

^b^ Significant difference between the first and the second bout in the same group at the same time point.

### Results

No differences in physical characteristics at baseline and at week 6 were observed between the 2 groups. All muscle performance measurements (except concentric torque) were modified significantly after the first bout of exercise indicating muscle damage only in the stair descending group. In contrast, no significant disturbances in muscle performance were observed after the last bout of exercise in either group (Table 1). After the six weeks of exercise, both training regimes increased muscle strength as determined by the assessment of isometric, concentric, and eccentric torque (although non-significantly in some cases due to the low number of participants). However, resting isometric torque increased significantly more after training in the eccentric group than the concentric group. The partial eta-square values for eccentric torque, concentric torque, isometric torque, ROM, DOMS and CK were 0.70, 0.16, 0.41, 0.09, 0.12 and 0.22, respectively.

### Discussion

In the present study, we used a mild protocol of stair descending exercise (a common daily activity) performed on the novel SmartEscalator device and we reported that it is capable to increase considerably muscle strength in elderly chronic heart failure patients. A limitation of the present study is the lack of a pure control group.

 The average heart rate for the descending group was lower during the last minute of exercise compared to the ascending group (98 ± 5 beats·min^-1^ vs. 139 ± 7 beats·min^-1^, respectively). In addition, based on the Borg rating (Borg 1970), average perceived exertion was lower during the last minute for the descending group compared to the ascending group (8.1 ± 1.3 vs. 13.6 ± 1.9, respectively). Indeed, even if human muscles do more positive than negative work during everyday activities [4], eccentric exercise has been repeatedly reported to induce less cardiovascular stress [5,6], and less fatigue [5-7], thus, it seems more appropriate for elderly and chronic heart failure patients.

 As expected [8], muscle damage appeared after the first bout of stair descending exercise. However, this muscle dysfunction disappeared after the last bout of exercise, indicating that adaptations took place in skeletal muscle of individuals participated in the stair descending protocol. After 6 weeks of stair exercise, the descending group (eccentric-biased activity) increased baseline eccentric torque by 12.3% whereas the ascending group (concentric-biased activity) increased concentric torque by 9.6%. Moreover, baseline isometric torque increased by 8.8% in the descending group and by 5.9% in the ascending group. Greater but qualitatively similar changes in muscle torque have been previously reported by our group using a pure-eccentric protocol of maximum effort [2,9].

 The loss of muscle strength that appears with aging is a major societal and economic challenge for the countries [10]. The economic burdens and social problems of aging and disease population deserve more attention than it has received [10]. Although healthier than previous generations, tomorrow’s elderly will ultimately require complex care for chronic disorders and multi-morbid states. Today, it is well-established that older individuals can gain a lot by keeping their muscle strength and functional performance [11]. In addition, muscle strength training improves quality of life, psychological well being and clinical parameters in patients with chronic heart failure [12].

 The present findings are of clinical importance for improving the quality of life in the elderly and/or diseased individuals. These people require a level of fitness to: (i) enable performing daily activities without undue fatigue; (ii) develop a reserve of energy for pleasure; (iii) make a faster and more complete recovery after debilitating illness; and (iv) promote a sense of personal well-being and zest for living.

 Muscle mass and strength is reduced due to aging or to physical inactivity-accompanying diseases [13]. Indeed, sarcopenia is a major factor contributing to decreased functional independence and mobility [14], while autonomy in daily living is the most important goal for the elderly [15]. Stair descending exercise appears to be a pleasant and mild activity that can be easily followed by the elderly or people with impaired endurance (i.e., diseased individuals). In addition, despite the comparatively little effort of stair descending exercise compared to stair ascending exercise, changes in muscle strength are similar or even greater. It is also noteworthy, that stair descending is a hard everyday obstacle for the elderly and physically disabled people. Therefore, the use of this type of exercise activity is particularly relevant to their motor needs and helps these people overcome a daily challenge for them. People who wish to increase their muscle strength may be benefited by participating in activities with strong eccentric component, such as stair descending. The results of this short scale study need to be confirmed in a larger scale study employing individuals suffering from other disease states (such as diabetes, rheumatoid arthritis, muscle atrophy) and measuring other health risk markers (such as blood lipid profile, blood pressure, insulin resistance) and quality of life.

## Competing interests

The authors declare that they have participated in the invention, design and construction of the SmartEscalator device. The device has been constructed for research use only.

## Authors’ contributions

TAA and PG contributed to the conception and the design of the study, performed the acquisition of data, drafted the article for important intellectual content. PV and KA contributed to the conception of the study, contributed to the analysis and interpretation of data, drafted and revised the article for important intellectual content. ML contributed to the design of the study, performed the acquisition of data, and drafted the article for important methodological and intellectual content. GGV performed the acquisition of data, and drafted the article for important intellectual content. NMG and VIS contributed to the design of the study and revised it critically. All authors read and approved the final manuscript.
